# Artificial intelligence hallucinations in large language models for medical education and healthcare: a scoping review of detection, mitigation, and governance

**DOI:** 10.1080/10872981.2026.2756806

**Published:** 2026-11-06

**Authors:** Sara heydari, zohreh sadat alavi

**Affiliations:** a Department of Medical Education, Medical Education and Development Center, Shahid Sadoughi University of Medical Sciences, Yazd, Iran; b Medical Education and Development Center, Shahid Sadoughi University of Medical Sciences, Yazd, Iran

**Keywords:** Artificial intelligence hallucinations, large language models, generative artificial intelligence, medical education, healthcare

## Abstract

**Background and objective:**

Generative artificial intelligence (AI) and large language models (LLMs) are increasingly used in medical education and healthcare. Yet AI hallucinations-plausible but inaccurate, unsupported, or fabricated information or citations-may compromise educational integrity, clinical judgment, patient safety, and trust. This scoping review mapped evidence on their nature, detection and validation, mitigation, and educational and governance implications.

**Methods:**

This scoping review was conducted in accordance with the Joanna Briggs Institute methodology and informed by the Arksey and O’Malley framework. Reporting followed the PRISMA Extension for Scoping Reviews. PubMed/MEDLINE, Scopus, IEEE Xplore, and Web of Science were systematically searched for English-language sources published from January 1, 2018, to July 31, 2026. Study selection and data extraction were independently performed by two reviewers. Findings were synthesized using descriptive thematic analysis.

**Results:**

Of 2,051 identified records, 21 sources were included in the final synthesis after duplicate removal and screening. These included five empirical or model-evaluation studies and 16 review, conceptual, or opinion-based sources. The analysis identified five main themes and 13 subthemes: (1) conceptualization and manifestations of AI hallucinations in healthcare; (2) detection, assessment, and validation of hallucinations; (3) mitigation strategies for improving the reliability of generative AI outputs; (4) educational implications and critical AI literacy; and (5) governance, accountability, and trustworthy implementation in healthcare. Retrieval-augmented generation, structured prompt design, output-verification methods, and human oversight were among the most frequently identified approaches for reducing hallucination-related risks.

**Conclusion:**

AI hallucinations are not merely technical errors; they represent a multidimensional challenge to the safe and responsible use of LLMs in medical education and healthcare. Reducing their risks requires a multilayered approach that includes evidence-based validation, human oversight, critical AI literacy, redesign of educational assessment, and transparent governance and accountability frameworks.

## Introduction

Artificial intelligence (AI) is a transformative technology with considerable potential to influence diverse fields, including healthcare and medical education [1]. In healthcare, AI may create new opportunities to improve service delivery and education through supporting personalized learning, analyzing complex data, improving the accuracy of selected diagnostic processes, and facilitating data-informed decision-making [2]. In this context, the integration of large language models (LLMs) into medical education and clinical practice has attracted substantial attention from researchers and healthcare professionals. These models can process and synthesize large volumes of medical text, generate educational content, summarize scientific literature, and, in some contexts, support the organization of information related to disease pathophysiology, diagnostic pathways, and treatment options. However, the value and safety of these tools depend on the accuracy, verifiability, and contextual appropriateness of their outputs in educational and clinical settings.

Despite the potential benefits of generative AI, its safe and responsible implementation in the sensitive context of healthcare is challenged by a major concern known as ‘AI hallucination.’ AI hallucinations refer to the generation of information, reasoning, or citations that are linguistically fluent and seemingly convincing but inaccurate, unsupported, or inconsistent with reliable evidence [3]. This phenomenon may be associated with factors such as generating responses in the absence of sufficient knowledge or context, producing statements that appear plausible but are incorrect, and relying on outdated, inaccurate, or contradictory information in training data [4]. Because LLMs primarily operate by predicting linguistic patterns, the fluency and apparent coherence of a response do not necessarily indicate its scientific or clinical accuracy.

Although the term ‘hallucination’ has become common in the A I literature to describe erroneous or unsupported outputs generated by AI systems, there is no global consensus on the appropriateness of this terminology or its operational definition. Different researchers have conceptualized this phenomenon by emphasizing aspects such as fabricated content, factual errors, inconsistency with source material, faulty reasoning, or the generation of false citations. Some scholars describe AI hallucinations as situations in which a chatbot generates fictional or unfounded content; however, the scope of this phenomenon extends beyond simple fabrication and may include factual, reasoning, temporal, and citation-related errors [5]. This conceptual heterogeneity makes it difficult to compare findings across studies, estimate the prevalence of hallucinations, and assess the effectiveness of mitigation strategies.

The consequences of AI hallucinations in healthcare can be serious. Providing inaccurate information about diagnosis, treatment, medication dosage, drug interactions, or care pathways may mislead users and, when used without appropriate expert review and oversight, may threaten patient safety. In addition, LLMs may generate citations with apparently accurate bibliographic details that are fabricated or refer to real but irrelevant sources. Such seemingly credible citations raise important concerns regarding source validity, the authenticity of bibliographic data, and the trustworthiness of AI chatbot outputs [6]. In medical education, uncritical use of generative AI responses may also lead to the dissemination of inaccurate knowledge, weaken learners’ skills in searching for and appraising evidence, and foster excessive reliance on technology.

The precise mechanisms underlying AI hallucinations are not yet fully understood. Some developers and researchers attribute these flawed outputs to a mismatch between users’ expectations and the actual capabilities of chatbots, limitations in training data, and issues related to machine-learning model training. Other explanations may relate to the way these systems process linguistic patterns, their lack of genuine understanding of causal and temporal relationships, and their treatment of bibliographic data as text that can be generated and manipulated [6]. Therefore, generated responses may initially appear logical and credible but can be misleading in practice; they should not serve as the basis for educational, research, or clinical decisions without independent validation [3].

As AI is rapidly integrated into various aspects of healthcare delivery, its expansion into medical education also appears inevitable [7]. This intersection has stimulated extensive discussion regarding the role of AI in learning, professional judgment, learner assessment, and the future of medical education. Generative AI may increase access to feedback, practice opportunities, and learning resources; however, it may also bypass cognitive processes that are essential for the development of clinical reasoning. Furthermore, the integration of generative AI into medical education should be designed in a way that preserves opportunities for ‘safe failure,’ enabling learners to test their clinical reasoning in a supportive environment, correct errors through feedback, and recognize their knowledge gaps without allowing AI to replace critical thinking and cognitive effort [8].

Accordingly, educating learners and faculty members to use AI critically and responsibly, evaluate the validity of AI-generated responses, verify sources, recognize model limitations, and maintain human oversight is essential. The safe deployment of these tools also requires transparent mechanisms for validation, accountability, privacy protection, bias mitigation, and responsible governance. Despite the rapid growth of research on generative AI, evidence on the nature of LLM hallucinations, methods for their detection and validation, mitigation strategies, and their educational and governance implications in healthcare remains scattered.

Therefore, this scoping review aimed to identify, map, and synthesize the available evidence on AI hallucinations in LLMs within medical education and healthcare. It focuses on the nature and manifestations of hallucinations, methods for their detection and assessment, mitigation strategies, and the educational, ethical, and governance considerations associated with the use of LLMs.

## Methods

### Study design

This study was conducted as a scoping review to identify, map, and synthesize the available evidence on artificial intelligence (AI) hallucinations in generative AI models, particularly large language models (LLMs), in medical education and clinical/healthcare applications. The review was conducted in accordance with the Joanna Briggs Institute (JBI) methodology for scoping reviews [9], with the methodological framework proposed by Arksey and O’Malley informing the review process [10]. The review was reported in accordance with the PRISMA Extension for Scoping Reviews (PRISMA-ScR) guideline [11]. The study selection process is presented using a PRISMA 2020 flow diagram [12].

### Stage 1: identification of the review questions and objectives

The review questions and objectives were developed using the Population–Concept–Context (PCC) framework.

The primary review question was:

What evidence is available regarding the nature, challenges, consequences, mechanisms, detection and validation methods, and mitigation strategies for AI hallucinations in generative AI models used in medical education and healthcare?

The secondary review questions were:

1. How have hallucinations in LLMs and generative AI been defined and classified in educational and clinical settings?
2. What methods, metrics, and benchmarks have been reported for detecting, measuring, and validating hallucinations or assessing the reliability of model-generated outputs?
3. What technical, educational, and governance strategies have been proposed or evaluated to prevent, manage, and mitigate AI hallucinations?

The PCC elements were defined as follows:

- **Population:** No restrictions were applied regarding user groups. Eligible sources included those addressing medical and health professions students, faculty members, physicians, other healthcare professionals, or the use of generative AI models in healthcare delivery.
- **Concept:** AI hallucinations, fabricated or unsupported information, factual inaccuracies, evidence-inconsistent outputs, fabricated citations, and approaches for the detection, validation, prevention, and mitigation of these errors in generative AI and LLMs.
- **Context:** Medical education, health professions education, clinical learning, clinical decision-making, educational simulation, biomedical research, and healthcare services.

### Stage 2: development and implementation of the search strategy

A systematic search was conducted in PubMed/MEDLINE, Scopus, IEEE Xplore, and Web of Science. Studies published from January 1, 2018, to July 31, 2026, were considered. These databases were selected to capture literature across medicine, health professions education, engineering, technology, digital health, and clinical practice.

The search strategy combined controlled vocabulary and free-text terms related to three main concepts: [1] technology, including ‘Generative AI,’ ‘Large Language Model,’ ‘LLM,’ ‘ChatGPT,’ ‘GPT-4,’ and ‘Foundation Model’; [2] hallucinations and related errors, including ‘Hallucination,’ ‘Confabulation,’ ‘Fabricated Information,’ ‘Factuality,’ ‘Faithfulness,’ ‘False Citation,’ and ‘Fabricated Citation’; and [3] context of use, including ‘Medical Education,’ ‘Health Professions Education,’ ‘Healthcare,’ ‘Clinical Practice,’ ‘Medical Training,’ and ‘Biomedical.’

To enhance search comprehensiveness, the reference lists of included studies were manually screened, and citation tracking of key sources was conducted. The complete search strategies for all databases should be provided in a supplementary file.

The PubMed search strategy was as follows:

text

(("artificial intelligence hallucination*" OR "AI hallucination*" OR

"LLM hallucination*" OR "large language model hallucination*" OR

"generative AI hallucination*" OR "ChatGPT hallucination*" OR

"hallucination in large language models" OR confabulation OR

"fabricated information" OR "false citation*" OR "hallucinated citation*" OR

"fabricated citation*" OR "spurious citation*" OR "made-up citation*" OR

"invented fact*" OR "misinformation in AI" OR "factuality" OR "faithfulness")

AND

("artificial intelligence" OR AI OR "large language model*" OR LLM OR

"generative AI" OR ChatGPT OR GPT* OR "foundation model*"))

AND

(medicine OR medical OR "medical education" OR healthcare OR "health care" OR

clinical OR biomedical OR "health professions education" OR "medical training")

AND

("2018/01/01"[Date - Publication]: "2026/07/31"[Date - Publication])

AND English[Language]

### Stage 3: study selection

Eligible sources were English-language publications with accessible full texts that directly addressed AI hallucinations, fabricated or unsupported information, factual inaccuracies, fabricated citations, or the reliability of outputs generated by generative AI models in medical education, health professions education, clinical applications, or healthcare.

Eligible source types included empirical studies, model evaluations and validation studies, scoping and narrative reviews, conceptual papers, expert perspectives, and theoretical articles with substantive analytical content. Sources were excluded if they were unrelated to medicine, medical education, health professions education, clinical practice, or healthcare; did not directly address AI hallucinations or the reliability of generative AI outputs; were conference abstracts without full text; were very brief reports without extractable data; or did not provide sufficient information to address the review questions.

Reference management and duplicate removal were performed using EndNote version 20. Two reviewers independently screened titles and abstracts and subsequently assessed full texts for eligibility. Disagreements were resolved through discussion and consensus. When necessary, a third reviewer contributed to the final decision.

A total of 2,051 records were identified through database searching. After removal of 842 duplicates, 1,209 records remained for title and abstract screening. At this stage, 1,140 records were excluded because they were not sufficiently relevant to the review topic. Thus, 69 full-text reports were assessed for eligibility.

Of the 69 full-text reports assessed, 48 were excluded: 19 because they did not directly focus on hallucinations or fabricated outputs; 16 because they were conducted in non-medical or otherwise inappropriate contexts; 8 because their source type or format was not suitable for extraction of the required data; and 5 because they did not provide sufficient extractable information. Ultimately, 21 sources were included in the final synthesis, comprising 5 empirical studies and 16 review, conceptual, or opinion-based sources (Figure 1).

Sources were not excluded solely because of incomplete methodological reporting, as the purpose of this scoping review was to map the breadth and nature of the available evidence. Nevertheless, methodological limitations and the nature of evidence were considered during interpretation of the findings.

**Figure 1.**
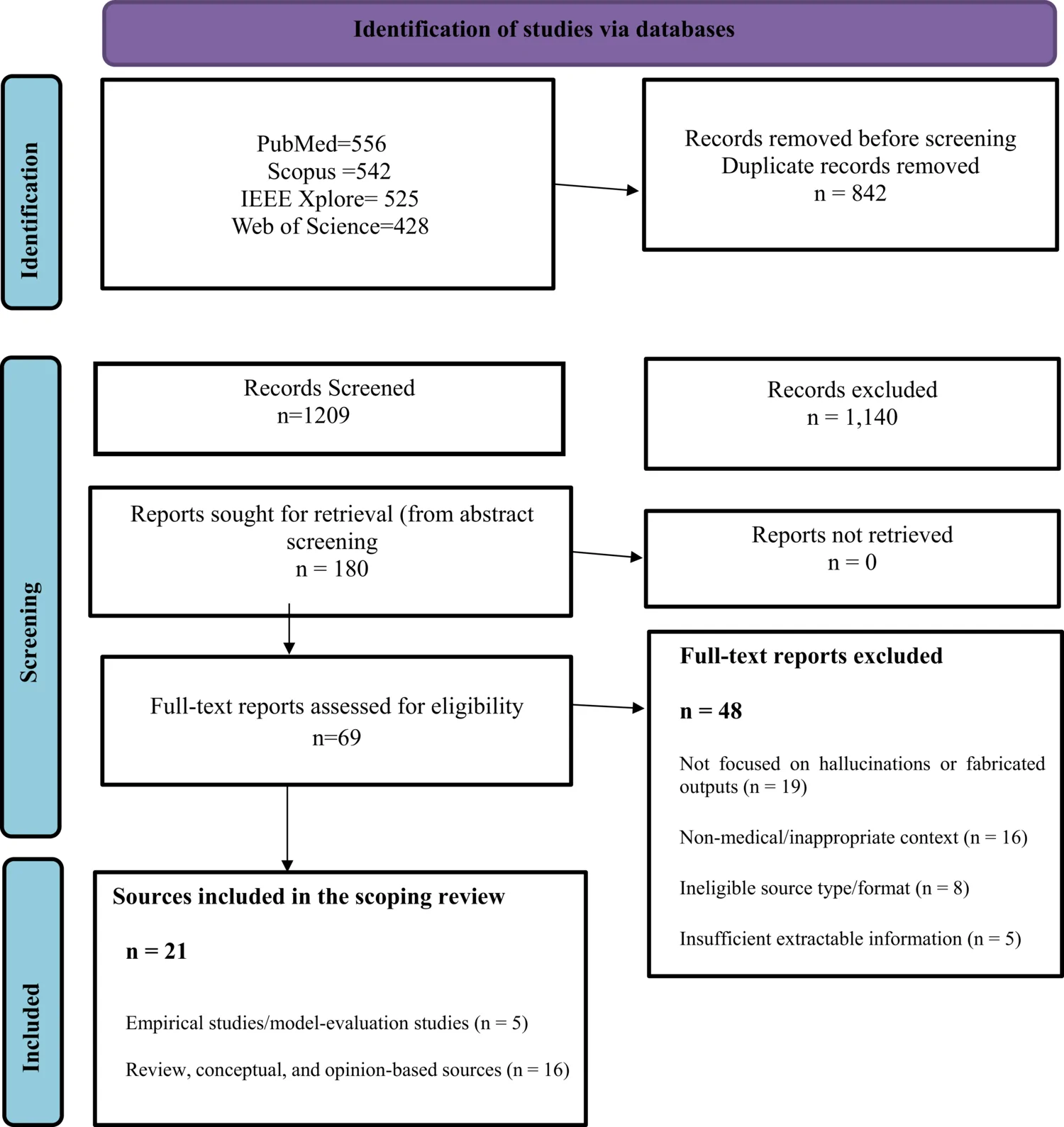
PRISMA 2020 flow diagram of the study identification, screening, eligibility assessment, and inclusion process.

### Stage 4: data charting and extraction

A data-charting form was developed based on the PCC framework, review objectives, and review questions. Extracted variables included author, year of publication, study title, study type and context, study aim, type of AI model, definition or classification of hallucination, principal hallucination-related findings, detection and evaluation methods, mitigation strategies, potential applications in medical education, educational and clinical implications, and reported limitations.

Data extraction was conducted independently by two reviewers. Extracted data were compared, and discrepancies were resolved through discussion and consensus. A third reviewer was consulted when disagreements persisted.

To avoid inappropriate aggregation of heterogeneous evidence, the included sources were grouped and presented in two separate tables. Table 1 presents the five empirical and model-evaluation studies that directly assessed performance, hallucinations, reliability, or mitigation approaches. Table 2 presents the 16 review articles, conceptual papers, and expert opinion sources that contributed to conceptual clarification, identification of challenges, mitigation approaches, educational implications, and governance considerations.

**Table 1.** Characteristics of empirical studies and model-evaluation studies (*n* = 5).

No.	Author, year	Study title	Study type and context	Study aim	Principal hallucination-related findings	Relevance to medical education	Reported limitations	Critical appraisal
1	Kim et al., 2025 [13]	*Medical Hallucination in Foundation Models and Their Impact on Healthcare*	Empirical study; foundation models and healthcare	To comprehensively evaluate medical hallucinations in foundation models, compare general-purpose and medical-specialized models, and examine mitigation strategies.	Gemini-2.5 Pro reportedly outperformed MedGemma in the study’s evaluation (97.9% vs. 61.9%); more than 64% of hallucinations were attributed to reasoning deficits rather than knowledge gaps; Chain-of-Thought prompting reduced hallucinations; 91.8% of physicians reported exposure to AI hallucinations.	Indicates that reliance on medical-specialized models alone may not ensure safety; educational programs should emphasize evaluation of reasoning and verification of AI outputs.	Moderate inter-rater agreement based on the Jaccard Index; limited qualitative sample of 20 cases from NEJM; physician survey mainly included participants from regions with strong AI infrastructure.	MMAT: 5/5 criteria met.
2	Li et al., 2025 [14]	*Comparative Analysis of Generative AI Systems in Solving Clinical Pharmacy Problems*	Empirical study; clinical pharmacy	To evaluate and compare eight generative AI systems across four clinical pharmacy scenarios.	DeepSeek-R1 achieved the highest reported performance scores; 75% of models overlooked contraindications; 90% lacked localization; only Claude-3.5-Sonnet identified a sex–diagnosis inconsistency.	Provides evidence supporting the need to train pharmacy and medical learners to critically assess AI-generated responses in medication-related scenarios.	Small case sample; incomplete coverage of pharmacy domains; results sensitive to prompt wording.	MMAT: 5/5 criteria met.
3	Sriram et al., 2025 [15]	*Context-Aware Retrieval-Augmented Generation for Artificial Intelligence in Urology*	Empirical study; urology	To evaluate context-aware retrieval-augmented generation (RAG) for reducing errors and improving the reliability of AI applications in urology.	The RAG system generated better responses than the baseline model in 88.5% of cases; RAG with Llama3-8B outperformed Llama3.3-70B without RAG; the system identified the fabricated disease ‘Aadhises’ and appropriately refused to provide an unsupported answer.	Offers an applied model for developing evidence-grounded educational tools in urology and reducing hallucinations.	Dependence on source-data quality; limited sample of 150 questions.	MMAT: 5/5 criteria met.
4	Xia et al., 2024 [16]	*MMed-RAG: Versatile Multimodal RAG System for Medical Vision Language Models*	Empirical study; medical vision–language models	To introduce a multimodal RAG system to improve alignment and reduce errors in medical vision–language models.	Reported a 43.8% improvement in factual accuracy; reduced Copy-Reference rate from 55% to 28.19%; reduced Over-Reliance rate from 43.31% to 8.38%; demonstrated superior performance across five medical datasets.	Supports the development of more reliable image-based medical education tools using AI-generated explanations.	High computational cost; dependence on data quality; implementation complexity.	MMAT: 5/5 criteria met.
5	Qian et al., 2025 [17]	*Towards Reliable Generative AI-Driven Scaffolding: Reducing Hallucinations and Enhancing Quality in Self-Regulated Learning Support*	Empirical study; self-regulated learning and educational scaffolding	To investigate approaches for improving the reliability of generative AI-driven educational support and reducing hallucinations.	A multi-agent architecture using GPT-4-Turbo achieved 93.1% accuracy in reliability evaluation; the LLM-as-a-Judge approach reportedly reduced hallucinations by 70%; four types of bias were identified in LLM judges.	Provides a framework for designing and evaluating AI-based educational scaffolds that may be adapted for medical education.	Stakeholders were not evaluated; no real-world implementation; dependence on a proprietary API.	MMAT: 5/5 criteria met.

**Table 2.** Characteristics of review, conceptual, and opinion-based sources (*n* = 16).

No.	Author, year	Study title	Study type and context	Study aim	Principal hallucination-related findings	Relevance to medical education	Reported limitations	Critical appraisal
1	Gadiko, 2024 [18]	*Understanding and Addressing AI Hallucinations in Healthcare and Life Sciences*	Theoretical/opinion paper; healthcare and life sciences	To examine the nature, causes, and mitigation strategies for AI hallucinations.	Classified hallucinations as input-conflicting, context-conflicting, and fact-conflicting; proposed RAG, Chain-of-Verification, and human-in-the-loop approaches; discussed FactScore, Med-HALT, and adversarial testing.	Provides a framework for identifying and mitigating hallucinations in AI-supported medical education.	Limited methodological detail; unclear sampling; incomplete reporting of tool validation; limited number of sources.	JBI: 5/6 criteria met.
2	Eysenbach, 2023 [19]	*The Role of ChatGPT, Generative Language Models, and Artificial Intelligence in Medical Education: A Conversation With ChatGPT and a Call for Papers*	Theoretical/opinion paper; medical education	To discuss the role and capabilities of ChatGPT in medical education.	ChatGPT may generate patient scenarios, simulations, and assessments; hallucinations in scientific references were highlighted; response quality was influenced by prompt engineering; AI was described as a complement to educators rather than a replacement.	Provides direct insights into the opportunities and limitations of ChatGPT in medical education.	Non-empirical design; dependence on prompt formulation; rapidly evolving technology.	JBI: 6/6 criteria met.
3	Roustan and Bastardot, 2025 [20]	*The Clinicians’ Guide to Large Language Models: A General Perspective With a Focus on Hallucinations*	Theoretical/opinion paper; clinical setting	To provide a general perspective for clinicians on LLMs, with emphasis on hallucinations.	Hallucinations were linked to data quality, the autoregressive nature of models, and user input; potential consequences included misdiagnosis, inappropriate treatment, and cognitive bias.	Offers practical guidance for educating clinicians about the safe use of LLMs in clinical settings.	Non-empirical; no quantitative data; no evaluation of a specific model.	JBI: 6/6 criteria met.
4	Gondode et al., 2024 [21]	*Artificial Intelligence Hallucinations in Anaesthesia: Causes, Consequences and Countermeasures*	Theoretical/opinion paper; anaesthesia	To review causes, consequences, and countermeasures for AI hallucinations in anaesthesia.	Identified insufficient data, model complexity, and black-box systems as contributors; discussed diagnostic and medication errors, research bias, and legal concerns; emphasized diverse data, explainable AI, and human oversight.	Provides a conceptual framework for addressing AI hallucinations in anaesthesia education.	Non-empirical; no quantitative data; relatively concise discussion.	JBI: 6/6 criteria met.
5	Jeong et al., 2026 [22]	*Generative Artificial Intelligence and Large Language Models in Competency-Based Medical Education: Applications, Challenges, and Future Directions*	Narrative review; competency-based medical education	To review applications, challenges, and future directions of generative AI and LLMs in competency-based medical education.	Identified hallucinations as a major challenge; proposed RAG as a mitigation strategy; emphasized AI literacy for recognizing hallucinations and a comprehensive framework for reducing risks.	Directly relevant to competency-based medical education, learner assessment, and AI literacy.	Narrative design; English-language focus; rapidly changing technology; no appraisal of included studies.	JBI: 6/6 criteria met.
6	Templin et al., 2024 [23]	*Addressing 6 Challenges in Generative AI for Digital Health: A Scoping Review*	Scoping review; digital health	To identify and analyze major challenges of generative AI in digital health.	Hallucination was identified as a key challenge; mitigation approaches included expert-in-the-loop review, temperature adjustment, and knowledge bases.	Offers a framework for teaching and research in digital health concerning major risks of generative AI.	English-language restriction; rapidly evolving technology.	JBI: 6/6 criteria met.
7	Bélisle-Pipon, 2024 [24]	*Why We Need to Be Careful With LLMs in Medicine*	Theoretical/opinion paper; medicine and healthcare	To explain why caution is needed when using LLMs in medicine.	Critiqued the term ‘hallucination’; emphasized the absence of genuine understanding in LLMs, output non-reproducibility, and the need for human oversight and appropriate regulation.	Supports education on intrinsic limitations of LLMs and the importance of professional caution.	Based on expert opinion; no quantitative data; limited practical mitigation detail.	JBI: 6/6 criteria met.
8	Shah et al., 2024 [25]	*Large Language Model Prompting Techniques for Advancement in Clinical Medicine*	Theoretical/opinion paper; clinical medicine	To review prompting techniques for improving LLM applications in clinical medicine.	Identified hallucinations as a major challenge; linked them to ambiguous prompts and limitations of training data; discussed zero-shot, one-shot, and few-shot prompting techniques.	Provides practical content for teaching prompt engineering and responsible use of LLMs in medicine.	Non-empirical; no quantitative data; limited clinical focus.	JBI: 6/6 criteria met.
9	Benavent et al., 2025 [26]	*RAGing Ahead in Rheumatology: New Language Model Architectures to Tame Artificial Intelligence*	Narrative review; rheumatology	To examine new LLM architectures and RAG approaches for improving reliability in rheumatology.	Identified hallucinations as a major challenge; proposed RAG as the main mitigation strategy and Chain-of-Verification as a complementary approach; discussed transparency and privacy benefits.	Provides a conceptual RAG framework for educating rheumatologists and researchers about reliable LLM use.	Non-empirical; limited rheumatology-specific evidence; infrastructure costs.	JBI: 6/6 criteria met.
10	Miao et al., 2024 [27]	*Integrating Retrieval-Augmented Generation With Large Language Models in Nephrology: Advancing Practical Applications*	Review/conceptual paper; nephrology	To examine integration of RAG with LLMs and its practical applications in nephrology.	Reported a high proportion of fabricated or incomplete references in LLM outputs; presented RAG as a primary strategy for hallucination reduction and Chain-of-Thought as a complementary approach.	Provides a practical RAG-oriented framework for educating nephrologists and researchers about LLM use.	Dependence on data quality; technical complexity; retrieval errors; need for further validation.	JBI: 6/6 criteria met.
11	Nakamura et al., 2026 [28]	*Generative AI in Medicine and Healthcare: A Comprehensive Review of Foundational Technologies, Clinical Applications, and Future Perspectives*	Comprehensive review; medicine and healthcare	To review foundational technologies, clinical applications, and future perspectives of generative AI.	Identified hallucination as a major challenge for LLMs; proposed RAG and human-in-the-loop approaches; discussed image hallucinations and generative imaging models.	Provides a broad overview for educating clinicians and researchers about generative AI technologies and limitations.	Non-empirical; limited new quantitative evidence; emphasis on emerging technologies.	JBI: 6/6 criteria met.
12	Meskó and Topol, 2023 [29]	*The Imperative for Regulatory Oversight of Large Language Models (or Generative AI) in Healthcare*	Theoretical/opinion paper; AI governance in healthcare	To explain the need for regulatory oversight of LLMs and generative AI in healthcare.	Identified hallucinations as a serious risk; discussed incomplete data, probabilistic model behavior, and lack of context; emphasized privacy, accountability, bias, and explainability.	Relevant to educating physicians, developers, and policymakers about responsible AI governance.	Non-empirical; no quantitative data; focus on the United States regulatory environment.	JBI: 6/6 criteria met.
13	Hatem et al., 2023 [30]	*A Call to Address AI* *‘Hallucinations’ and How Healthcare Professionals Can Mitigate Their Risks*	Theoretical/opinion paper; healthcare and health professions	To discuss hallucination-related risks and mitigation strategies for healthcare professionals.	Proposed ‘AI misinformation’ as a potentially more precise term; discussed fabricated scientific references; recommended education, verification, error reporting, and diverse data.	Highlights the role of healthcare professional education and human oversight in safe AI implementation.	Non-empirical; no quantitative data; emphasis on conceptual and linguistic issues.	JBI Text and Opinion: 6/6 criteria met.
14	Thompson et al., 2025 [31]	*Artificial Intelligence Use in Medical Education: Best Practices and Future Directions*	Narrative review; medical education	To review best practices and future directions for AI use in medical education.	Identified hallucinations as a major concern; cited fabrication of scientific references; emphasized careful output review, clear guidelines, specialized tools, and physician involvement.	Supports development of best-practice guidance for ethical and effective AI use in medical education.	Non-empirical; no quantitative data; focus limited to medical education.	JBI: 6/6 criteria met.
15	Preiksaitis and Rose, 2023 [7]	*Opportunities, Challenges, and Future Directions of Generative Artificial Intelligence in Medical Education: Scoping Review*	Scoping review; medical education	To explore opportunities, challenges, and future directions of generative AI in medical education.	Identified hallucination as a major challenge; discussed fabricated references, the probabilistic nature of model outputs, AI literacy, critical thinking, and human–AI interaction.	Provides a conceptual framework for AI literacy, assessment redesign, and critical thinking in medical education.	English-language restriction; limited time frame; scarcity of empirical studies.	JBI: 6/6 criteria met.
16	Berry et al., 2025 [32]	*Utilizing Large Language Models for Gastroenterology Research: A Conceptual Framework*	Review/conceptual framework; gastroenterology research	To develop a conceptual framework for using LLMs in gastroenterology research.	Identified hallucination as a major challenge; discussed RAG, fine-tuning, calibration, out-of-distribution detection, reinforcement learning from human feedback, and prompt optimization.	Provides a stepwise conceptual framework for educating gastroenterology specialists about safe LLM use.	Non-empirical; no quantitative data; not validated in real-world settings.	JBI: 6/6 criteria met.

### Stage 5: critical appraisal of included sources

Although critical appraisal was not used as an exclusion criterion in this scoping review, it was conducted to describe methodological limitations and support cautious interpretation of the findings. No study was excluded solely on the basis of appraisal results.

The five included empirical studies [13–17]were appraised using the 2018 version of the Mixed Methods Appraisal Tool (MMAT) [33]. Review articles were appraised using JBI critical appraisal checklists appropriate to the source type [34]. Theoretical, conceptual, and opinion-based articles were appraised using the JBI Critical Appraisal Checklist for Text and Opinion [35].

Two reviewers independently conducted the appraisal process, and disagreements were resolved through consensus. Appraisal results are reported in Tables 1 and 2. MMAT and JBI scores are presented as the number of criteria met and should not be interpreted as definitive overall quality rankings.

### Stage 6: data synthesis, analysis, and reporting

A descriptive thematic content analysis was used to synthesize the extracted data. First, all extracted information was reviewed repeatedly. Meaning units related to the nature and manifestations of hallucinations, mechanisms of occurrence, detection and validation approaches, mitigation strategies, educational and clinical implications, and ethical and governance considerations were identified and coded.

Initial codes were generated inductively and refined through constant comparison, iterative review, and discussion among the research team. The final analytical framework comprised five main themes and 13 subthemes:

1. Conceptualization and manifestations of AI hallucinations in healthcare;
2. Detection, assessment, and validation of hallucinations;
3. Mitigation strategies for improving the reliability of generative AI outputs;
4. Educational implications and critical AI literacy;
5. Governance, accountability, and trustworthy implementation in healthcare.

Empirical studies and review, conceptual, and opinion-based sources were initially described separately because of their different forms of evidence. Their findings were subsequently integrated through narrative synthesis. Theoretical and opinion-based sources were used to clarify concepts, identify knowledge gaps, and describe educational and governance implications; they were not used as the basis for conclusions regarding the effectiveness of interventions.

Following theme refinement, an integrative conceptual framework was developed to visually represent the relationships among the five themes and their contribution to trustworthy implementation of generative AI in medical education and healthcare.

## Results

A total of 21 sources were included in the final synthesis. Of these, five were empirical studies or model-evaluation studies that directly examined hallucinations, output reliability, or hallucination-mitigation approaches in generative AI and large language models (LLMs) in healthcare-related contexts [13–17]. The remaining 16 sources consisted of scoping reviews, narrative reviews, conceptual papers, and expert opinion articles that contributed contextual, theoretical, educational, and governance-related evidence [7,18–32]. The characteristics of the 21 included sources are presented in Tables 1 and 2.

Given the heterogeneity of the included sources in terms of study design, AI model, healthcare setting, outcome measures, and definitions of hallucination, quantitative pooling was not undertaken. Instead, a descriptive thematic synthesis was conducted. The analysis identified five main themes and 13 subthemes: [1] conceptualization and manifestations of AI hallucinations in healthcare; [2] detection, assessment, and validation of hallucinations; [3] mitigation strategies for improving the reliability of generative AI outputs; [4] educational implications and critical AI literacy; and [5] governance, accountability, and trustworthy implementation in healthcare.

**Theme 1. Conceptualization and manifestations of AI hallucinations in healthcare**

This theme describes how hallucinations were conceptualized in the included sources, how they were distinguished from general model errors, and how they may manifest in healthcare settings. The sources consistently characterized hallucinations as outputs that may appear coherent and convincing but are inaccurate, unsupported, inconsistent with the provided context, or based on fabricated references. In healthcare, such outputs are particularly concerning because their apparent plausibility may obscure their lack of factual or clinical validity.

**1.1. Hallucinations as threats to factual accuracy and reliability**

The included sources described hallucinations as a threat to the factual accuracy, reliability, and trustworthiness of AI-generated medical information. Hallucinations included unsupported diagnostic or therapeutic statements, inaccurate medication-related information, fabricated mechanisms of disease, and nonexistent or misleading scientific references. Although not all inaccurate outputs necessarily result in patient harm, the sources emphasized that unverified AI-generated information may be consequential when used in clinical reasoning, education, research, or healthcare decision-making [13,18,20,21,24,29].

**1.2. Types and patterns of hallucinations**

The evidence suggested that hallucinations may occur in several interrelated forms. These included factual hallucinations, in which model-generated information conflicts with established scientific or clinical knowledge; context-conflicting hallucinations, in which responses are inconsistent with the clinical scenario or information provided by the user; input-conflicting outputs, which may arise in response to ambiguous, incomplete, or misleading prompts; and citation hallucinations, involving fabricated, inaccurate, or irrelevant references. This classification highlights that hallucinations are not limited to a single technical failure but may emerge at different stages of information processing and response generation [13,18,20,21,23,25].

**1.3. High-risk clinical manifestations**

High-risk manifestations of hallucinations included incorrect treatment or medication recommendations, failure to identify contraindications, unsupported diagnostic suggestions, fabricated clinical explanations, and misleading content in clinical documentation or evidence summaries. The potential consequences may be amplified when users lack sufficient expertise to identify inaccuracies or when they rely on the apparent confidence and fluency of AI-generated responses. Empirical and conceptual sources particularly emphasized the importance of caution in complex, time-sensitive, and high-stakes clinical contexts [13,20,21,27,28,30].

**Theme 2. Detection, assessment, and validation of hallucinations**

This theme addresses how hallucinations can be identified, measured, and validated. The findings indicated that no single metric or method is sufficient to detect all forms of hallucination. Instead, the included studies supported a multi-component evaluation approach that combines task-specific performance assessment, comparison with reliable evidence sources, expert review, and testing under clinically challenging conditions.

**2.1. Benchmarking and task-specific assessment of factuality**

Several studies emphasized the need for structured clinical scenarios, benchmark datasets, verified reference answers, and performance indicators that extend beyond general accuracy. Relevant measures included factual accuracy, citation validity, consistency with clinical context, ability to recognize uncertainty, and appropriate refusal to answer unsupported or fabricated questions. For example, empirical studies evaluated AI models using clinical pharmacy cases, urology questions, multimodal medical datasets, and educational scaffolding tasks. These findings suggest that evaluation should be tailored to the intended clinical or educational use of the model rather than relying on general-purpose benchmarks alone [13–17,23,25].

**2.1. Expert validation and context-sensitive testing**

The sources consistently highlighted the importance of expert review in detecting hallucinations and assessing the clinical relevance of AI-generated outputs. Expert validation may include checking the factual correctness of content, verifying citations, assessing consistency with patient-specific information, and determining the potential severity of an incorrect response. In addition, models should be tested under conditions resembling real clinical complexity, such as incomplete information, conflicting data, multimorbidity, ambiguous prompts, and tasks involving temporal or causal reasoning. Such testing is particularly important because a model may perform acceptably on simple questions while producing unsafe or unreliable outputs in more complex contexts [13,20,21,24,26–28,30,32].

**Theme 3. Mitigation strategies for improving the reliability of generative AI outputs**

This theme includes technical and human-centered approaches intended to reduce the likelihood or impact of hallucinations. Across the included sources, mitigation was not described as the complete elimination of hallucinations; rather, it was framed as improving the grounding, transparency, verifiability, and safety of generated outputs.

**3.1. Evidence grounding through retrieval-augmented generation**

Retrieval-augmented generation (RAG) was the most frequently discussed technical strategy for reducing hallucinations. RAG connects the model to external, curated, and up-to-date knowledge sources, such as clinical guidelines, medical databases, peer-reviewed literature, and institutional documents. By supplying relevant evidence during response generation, RAG may improve factual grounding, facilitate citation checking, and reduce reliance on the model’s internal statistical associations. The empirical studies in urology and multimodal medical AI, as well as several conceptual and review sources, reported that RAG-based approaches may improve response quality and reduce unsupported outputs; however, their performance remains dependent on the quality, relevance, completeness, and currency of retrieved evidence [15,16,18,26,27,32].

**3.2. Prompt design and automated verification approaches**

Structured prompt design, Chain-of-Thought prompting, Chain-of-Verification, self-review procedures, and automated output-evaluation approaches were identified as complementary mitigation strategies. These methods aim to guide the model toward more systematic reasoning, encourage verification of generated claims, identify uncertainty, or compare outputs against predefined criteria. The empirical study on educational scaffolding also suggested that multi-agent approaches and automated LLM-based evaluation may improve reliability; however, it also identified potential bias in LLM judges. Therefore, these approaches should be regarded as supportive safeguards rather than independent substitutes for expert validation [13,17,18,25].

**3.3. Human-in-the-loop implementation**

Human oversight was consistently identified as a necessary component of safe implementation. In healthcare settings, AI-generated content should support, rather than replace, professional judgment. Clinicians, educators, or other qualified users should verify the accuracy of outputs, assess the appropriateness of recommendations for the individual context, evaluate citations, and determine the potential consequences of error before using AI-generated information in clinical or educational decisions. Human-in-the-loop implementation was therefore presented as both a technical safety measure and a professional responsibility [20,21,24,28,30].

**Theme 4. Educational implications and critical AI literacy**

This theme demonstrates that hallucinations are not solely technical problems; they are also educational challenges. The included sources emphasized that learners and educators require competencies that enable them to use generative AI critically, interpret its limitations, and distinguish fluent responses from accurate and evidence-based answers.

**4.1. Critical AI literacy and appraisal of AI-generated outputs**

The findings indicated that medical students, residents, faculty members, and healthcare professionals should develop critical AI literacy. This competence includes recognizing potentially unreliable responses, verifying sources, understanding the limitations of probabilistic language models, identifying contexts in which hallucinations are more likely, and evaluating the difference between persuasive language and clinically valid information. Educational programs should therefore move beyond teaching how to use AI tools and include instruction on how to question, validate, and appropriately limit their use [7,14,19,22,31].

**4.2. Safe educational integration and assessment redesign**

Generative AI may support the development of clinical scenarios, formative feedback, simulation activities, question generation, explanations of concepts, and self-regulated learning. However, the included sources emphasized that AI-generated educational material should be reviewed by qualified educators or subject experts before use. The findings also highlighted the need to reconsider assessment approaches, as uncritical reliance on AI-generated content may encourage superficial learning, reduce independent reasoning, facilitate academic misconduct, and introduce inaccurate knowledge into clinical training. Accordingly, safe integration of AI in medical education requires clear guidance, transparent disclosure of AI use, expert review of generated content, and assessment designs that continue to evaluate authentic clinical reasoning and professional competence [7,14,17,19,22,24,30,31].

**Theme 5. Governance, accountability, and trustworthy implementation in healthcare**

This theme addresses the organizational, ethical, and regulatory conditions required for responsible use of generative AI in medical education and healthcare. The findings suggested that technical mitigation alone is insufficient; trustworthy implementation also requires transparency, clear accountability, protection of sensitive data, and attention to equity and patient safety.

**5.1. Transparency, traceability, and disclosure of limitations**

The sources emphasized that users should know when content has been generated or supported by AI, what information sources were used, whether citations are verifiable, and what limitations may affect the reliability of the output. Transparency and traceability were presented as prerequisites for informed trust rather than unquestioning trust. Systems should therefore facilitate access to supporting evidence, disclose uncertainty when appropriate, and avoid presenting unsupported content with unwarranted confidence [20,23,24,28,29].

**5.2. Accountability, human oversight, and regulation**

The included sources identified ambiguity regarding responsibility for AI-related errors as a major challenge. Accountability should not remain unclear between developers, healthcare organizations, educators, and end users. Governance frameworks should specify the intended use of AI tools, define the level of required human review, establish mechanisms for auditing and reporting errors, and clarify professional and institutional responsibilities. Regulatory oversight was particularly emphasized for high-risk clinical applications in which hallucinated outputs may influence diagnosis, treatment, or patient communication [21,24,28–30].

**5.3. Equity, privacy, and safety in implementation**

The sources also highlighted the potential interaction between hallucinations and broader concerns about data representativeness, algorithmic bias, privacy, and unequal access to reliable AI tools. Biased or non-representative data may increase the likelihood of inaccurate outputs for underrepresented groups, while inadequate privacy safeguards may expose sensitive patient information. Therefore, implementation strategies should incorporate equity-oriented design, data protection, continuous monitoring, and context-specific safety evaluation to avoid worsening existing disparities or introducing new harms [23,24,29].

Based on the thematic synthesis, we proposed the REMEG-Med conceptual framework for trustworthy implementation of generative AI in medical education and healthcare (Figure 2). The framework comprises five interconnected domains: Recognize AI hallucinations; Evaluate and validate generated outputs; Mitigate risks through technical and human-centered safeguards; Educate learners and faculty members to develop critical AI literacy; and Govern AI use through transparency, accountability, equity, privacy, and safety mechanisms. The framework suggests that hallucination management cannot be addressed solely through technical model improvements and requires continuous interaction among evidence-based validation, human oversight, educational design, and institutional governance. REMEG-Med is a conceptual framework derived from this scoping review and requires future validation in real-world educational and clinical settings.

### The REMEG-Med Framework

*A conceptual framework for trustworthy implementation of generative AI in medical education and healthcare.*

**Figure 2.**
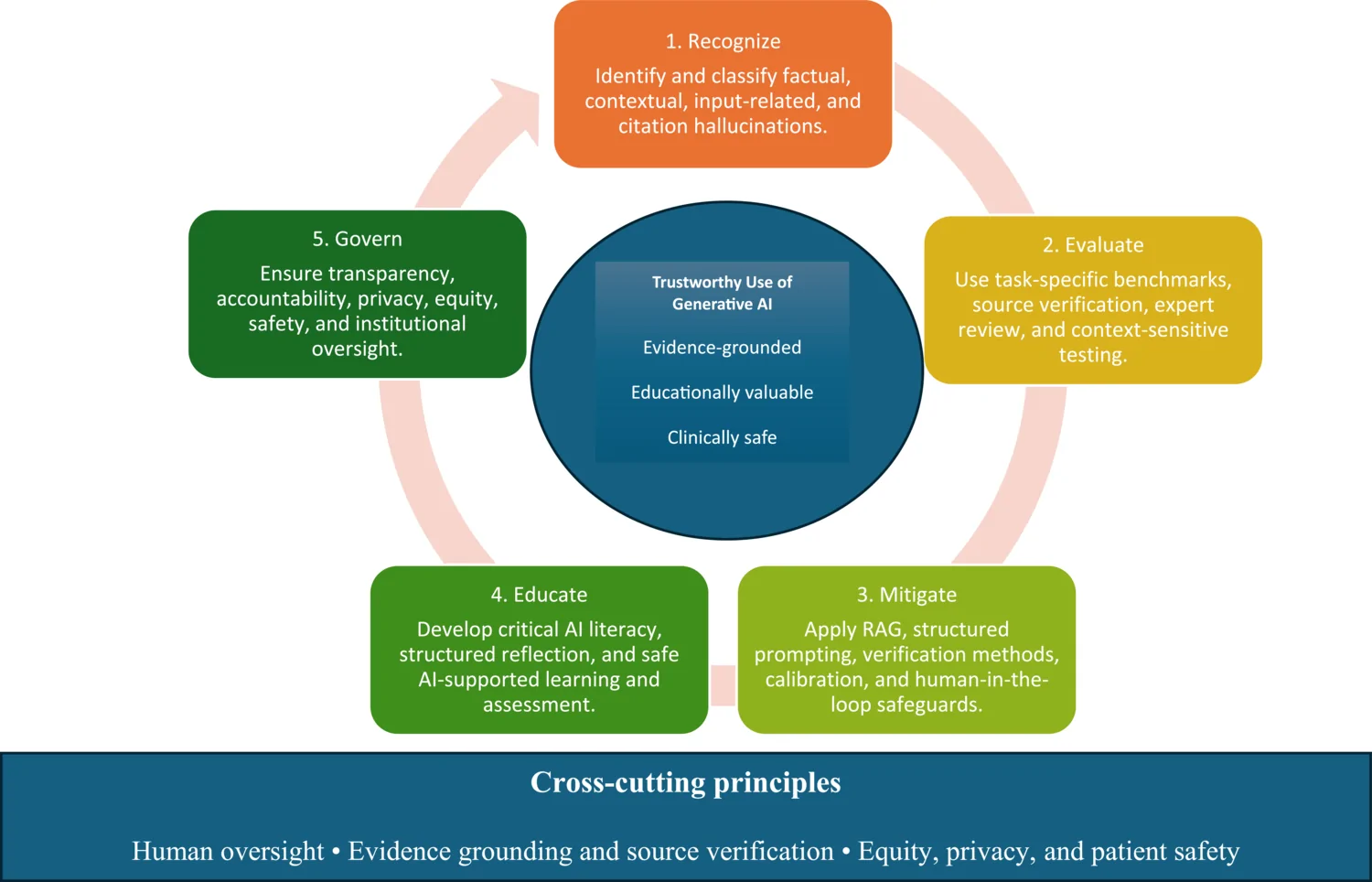
The REMEG-Med framework for trustworthy implementation of generative AI in medical education and healthcare.

## Discussion

This scoping review mapped the available evidence on AI hallucinations in large language models (LLMs) and other generative AI tools used in medical education and healthcare. Of the 21 included sources, five were empirical or model-evaluation studies, while 16 provided review, conceptual, or opinion-based evidence. The synthesis identified five interconnected areas: conceptualization and manifestations of hallucinations in healthcare; detection and validation; mitigation strategies and reliability enhancement; educational implications and critical AI literacy; and governance and accountability for trustworthy implementation. Overall, the findings indicate that AI hallucinations are not isolated technical errors; they represent a challenge to information validity, learning quality, patient safety, and trust in AI-supported healthcare.

The REMEG-Med framework integrates these findings into an actionable conceptual structure and highlights that trustworthy AI implementation requires coordinated technical, educational, professional, and institutional responses rather than isolated interventions.

### Nature and significance of AI hallucinations in medicine

The findings of this review suggest that AI hallucinations in medical contexts extend beyond simple factual errors. They may include unsupported diagnostic or therapeutic recommendations, inaccurate medication-related information, fabricated clinical explanations, responses inconsistent with the patient context, and fabricated citations. Because LLM outputs are often fluent, coherent, and apparently credible, users may accept them uncritically, particularly when they lack sufficient expertise or time to verify the information [13,18,20,21,24,29].

Kim et al. reported that more than 64% of the identified hallucinations were associated with reasoning limitations rather than knowledge deficits [13]. Although this finding should be interpreted cautiously because it was derived from a specific study and evaluation dataset, it shifts attention beyond merely expanding medical training data. It highlights the importance of designing models with assessable reasoning capabilities, testing them in complex scenarios, and validating their outputs. For medical education, this finding is important because access to extensive medical knowledge does not, by itself, guarantee the accuracy of AI-generated reasoning or the clinical appropriateness of a response.

The typology of hallucinations identified in this review—including factual, context-conflicting, input-conflicting, and citation hallucinations—provides a practical framework for analyzing AI-generated outputs. This typology may be incorporated into educational activities, simulation scenarios, and assessment tasks. For example, learners may be asked to examine an AI-generated response in terms of factual accuracy, patient-context relevance, reasoning quality, and citation validity. At the same time, some sources cautioned against the anthropomorphic implications of the term ‘hallucination’ and emphasized that LLMs do not possess human understanding or an inherent commitment to truth. Accordingly, users should not equate linguistic fluency or apparent confidence with scientific validity [24].

The clinical implications are substantial. Included sources described risks such as inappropriate diagnosis, inaccurate treatment or medication recommendations, failure to identify contraindications, cognitive bias in clinical reasoning, and misleading clinical documentation [14,20,21,27,28,30]. These findings suggest that AI hallucinations should not be treated as a peripheral technical concern. Rather, they should be incorporated into medical education as part of patient safety, clinical reasoning, professional ethics, and evidence-informed use of digital technologies.

### Detection, assessment, and validation of hallucinations

This review found that detecting AI hallucinations in healthcare requires a multilayered approach. Automated metrics and standardized benchmarks may be useful for comparing models and identifying obvious errors; however, they are insufficient for assessing the clinical appropriateness of a response. Meaningful evaluation should include structured clinical scenarios, reliable reference answers, citation verification, sensitivity testing across prompts, and expert review [13–17,23,25].

The difficulty of identifying hallucinations is evident even among expert reviewers. Kim et al. reported moderate agreement among evaluators regarding hallucination type and clinical risk level [13]. This finding suggests that distinguishing between an incorrect answer, an incomplete answer, a contextually inappropriate response, and a genuinely unsafe response is not always straightforward. Therefore, education on AI hallucinations should extend beyond simple checklists and focus on clinical reasoning, evidence interpretation, patient-context analysis, and judgment regarding the potential consequences of error.

The included sources also indicated that model performance may appear acceptable in simple tasks but deteriorate when information is incomplete or conflicting, when multiple comorbidities are present, when prompts are ambiguous, or when tasks require causal or temporal reasoning [13,26–28,32]. Therefore, evaluation of AI tools for medical education and clinical practice should prioritize realistic and contextually complex scenarios. Assessments limited to simple right-or-wrong questions may underestimate the risks associated with real-world use.

### Mitigation strategies: from evidence grounding to human oversight

The findings support a combined approach to mitigating hallucinations. Retrieval-augmented generation (RAG) was the most frequently identified technical strategy. RAG connects an AI model to external and potentially verifiable sources, such as clinical guidelines, medical databases, scientific articles, or institutional documents, during response generation. This approach may increase the factual grounding and traceability of AI-generated content and facilitate source verification by users [15,16,18,26,27,32].

For example, Sriram et al. reported that a context-aware RAG system produced better responses than the baseline model in 88.5% of cases and appropriately declined to provide unsupported information about a fabricated disease [15]. Similarly, Xia et al. found that multimodal RAG improved factual accuracy and alignment-related outcomes in medical vision–language models [16]. Nevertheless, these findings should not be interpreted as evidence that RAG eliminates hallucinations. Its effectiveness depends on the quality, relevance, completeness, and currency of the retrieved information, and retrieval errors may themselves contribute to inaccurate outputs.

Complementary approaches include structured prompt engineering, Chain-of-Thought prompting, Chain-of-Verification, automated output review, model calibration, fine-tuning, out-of-distribution detection, and learning from human feedback [13,17,18,25,32]. These approaches may improve output reliability, but the current evidence does not support the superiority of a single strategy across all educational or clinical contexts. Hallucination mitigation should therefore be understood as a multilayered safety design rather than a one-time technical intervention.

Human oversight remains essential. Qualified users should verify content accuracy, evaluate citation validity, assess the relevance of recommendations to the patient context, and consider the potential consequences of error before using AI-generated outputs. This interpretation is consistent with our previous article on AI applications and ethical issues in emergency medicine, which emphasized transparency in model training, careful selection of unbiased training data, protection of patient privacy, and the retention of final treatment decisions by physicians [36]. Accordingly, generative AI should be positioned as a decision-support tool rather than an autonomous decision-maker [20–22,24,28,30].

Furthermore, to operationalize human-in-the-loop oversight efficiently during content and curriculum design, emerging paradigms such as hybrid AI-generation (Hybrid AIG) offer a promising avenue. By shifting the role of large language models from direct content generation-which inherently carries high hallucination risks-to structural template creation, human experts retain full control over populating and validating clinical and educational content. This collaborative architecture ensures that generative outputs remain strictly accurate while scaling efficiently for medical education [37,38]. Future mitigation frameworks should increasingly integrate such hybrid co-generation workflows to bridge the gap between technical scalability and clinical safety.

### Implications for medical education

The findings of this review indicate that the growing availability of generative AI requires purposeful adaptation of medical education. ChatGPT and similar tools may support the generation of patient scenarios, formative feedback, simulation activities, question development, conceptual explanations, and self-regulated learning. However, these same tools may generate inaccurate scientific content or fabricated citations. Their educational value therefore depends on the design of learning activities, the quality of faculty oversight, and learners’ ability to critically appraise AI-generated outputs [7,17,19,22,30,31].

AI literacy should be developed as a longitudinal competency within medical curricula. This competence includes understanding the general functioning and limitations of LLMs, recognizing high-risk situations, verifying information sources, comparing AI-generated responses with reliable evidence, identifying uncertainty, and reporting errors. Preiksaitis and Rose emphasized the importance of AI literacy, assessment redesign, and attention to human–AI interaction in medical education [7]. Thompson et al. similarly highlighted the need to preserve critical thinking through balanced AI integration [31].

Furthermore, the safe use of generative AI should be accompanied by structured reflective activities that enable learners to critically examine their experiences with AI, the reasoning underlying generated responses, the sources used, areas of uncertainty, and potential errors. Such an approach aligns with the role of reflective practice in strengthening clinical reasoning, professional development, and ethical decision-making in medical education [39].

Learner assessment should also adapt to the increasing availability of generative AI tools. Text-based assignments completed outside supervised settings may not accurately represent a learner’s independent clinical reasoning. In contrast, structured oral examinations, observed clinical encounters, case-based tasks, and activities that require learners to critique, correct, or reject AI-generated responses may better assess authentic reasoning and responsible technology use. The goal is not to exclude AI from medical education, but to create learning environments in which AI strengthens learning rather than replacing cognitive effort and professional judgment.

### Governance, accountability, and trustworthy implementation

The review findings indicate that technical safeguards alone are insufficient to manage hallucination-related risks. Responsible implementation of LLMs in medical education and healthcare requires transparency about model capabilities and limitations, traceability of supporting sources, clear expectations for human oversight, auditing mechanisms, error-reporting systems, and defined responsibilities for stakeholders [20,23,24,28,29].

The included sources emphasized that responsibility for AI-related errors should not remain ambiguous among developers, healthcare organizations, educators, and end users. Governance frameworks should specify the intended purpose of AI tools, define the level of required human review, establish mechanisms for auditing and reporting errors, and clarify professional and institutional responsibilities. Regulatory approaches should also account for characteristics unique to generative AI, including variable outputs, apparently credible but unsupported content, and potential use in unforeseen high-risk contexts [21,28–30].

Privacy, bias, and equity are integral to this issue. Biased or non-representative training data may increase the likelihood of inaccurate or inappropriate outputs for underrepresented populations. In addition, entering sensitive patient information into systems without clear data-protection policies may create ethical and legal concerns. Institutional policies should therefore govern data use, access to AI tools, safety evaluation processes, and accountability mechanisms through an equity-oriented and privacy-protective approach [22–24,29].

### Limitations and directions for future research

This scoping review has several limitations. First, the search was restricted to English-language sources and four databases; therefore, relevant evidence published in other languages or in grey literature may not have been identified. Second, the search was completed on July 31, 2026. Given the rapid evolution of generative AI technologies, more recent studies and tools may not be represented. Third, substantial heterogeneity in study designs, AI models, clinical scenarios, definitions of hallucination, and outcome measures precluded quantitative synthesis and direct comparisons of mitigation strategies. Fourth, most included sources were reviews, conceptual papers, or expert opinions; thus, the findings should primarily be interpreted as a map of available evidence and research gaps rather than definitive estimates of intervention effectiveness.

Future research should focus on developing standardized, context-sensitive measures for detecting AI hallucinations in medical education and clinical care. Experimental studies should compare RAG, automated verification methods, prompt-engineering approaches, multi-agent systems, and human oversight models using real-world scenarios and clearly defined educational or clinical outcomes. Longitudinal studies are also needed to examine how AI literacy develops among students and faculty members and how this literacy influences clinical reasoning, professional behavior, and patient safety. In addition, implementation research should investigate barriers and facilitators to responsible AI integration in medical schools and teaching hospitals.

### Practical implications

Several practical actions can be derived from this review. First, AI literacy should be integrated longitudinally into medical curricula rather than being limited to single educational sessions. Second, learners should have repeated opportunities to compare AI-generated outputs with reliable sources and identify factual, contextual, and citation-related errors. Third, faculty development is essential, as educators need the knowledge and skills required to evaluate and teach the responsible use of AI. Fourth, medical schools and healthcare organizations should establish clear policies regarding acceptable AI use in education, assessment, research, and clinical practice. These policies should include requirements for transparency, source verification, patient-data protection, human oversight, and error reporting.

## Conclusion

This scoping review shows that AI hallucinations represent a fundamental challenge to the safe and responsible integration of generative AI and large language models into medical education and healthcare. Hallucinations are not merely technical inaccuracies; they may generate plausible but unsupported clinical information, misleading reasoning, and fabricated citations that can compromise educational integrity, clinical judgment, patient safety, and trust.

The synthesis of 21 sources identified five interrelated priorities: understanding the manifestations of hallucinations; strengthening detection, assessment, and validation; implementing layered mitigation strategies; developing critical AI literacy in medical education; and establishing governance, accountability, and equity-oriented safeguards. Technical approaches such as retrieval-augmented generation, prompt optimization, verification methods, and model calibration may improve output reliability, but none can fully eliminate hallucinations. Human oversight and independent verification therefore remain essential.

Medical education should prepare learners and faculty members not only to use generative AI tools, but also to critically appraise their outputs, verify sources, recognize uncertainty, and respond appropriately to potential errors. Educational institutions and healthcare organizations should establish clear policies for AI use, assessment, privacy protection, transparency, error reporting, and professional accountability.

The objective should not be to exclude generative AI from medical education or healthcare, but to integrate it in ways that enhance learning and support clinical work without replacing clinical reasoning, ethical judgment, or professional responsibility. The proposed REMEG-Med framework provides a practical conceptual structure for integrating technical safeguards, critical AI literacy, human oversight, and institutional governance; however, it requires further validation and refinement in real-world educational and clinical settings.

## Supplementary Material

Supplementary_File_1.pdf Supplementary_File_1.pdf

Supplementary_Information_2.pdf Supplementary_Information_2.pdf

## Data Availability

The datasets generated and analyzed during the current study are available from the corresponding author on reasonable request.

## Supplementary information

The supplementary material includes Supplementary File 1, containing the full electronic search strategies for PubMed/MEDLINE, Scopus, IEEE Xplore, and Web of Science; and Supplementary File 2, containing the PRISMA-ScR checklist.
