# Supplementary material for: Artificial intelligence hallucinations in large language models for medical education and healthcare: a scoping review of detection, mitigation, and governance
Source: Med Educ Online. 2026 Nov 6;31(1):2756806. doi: 10.1080/10872981.2026.2756806 (PMC13757853; doi:10.1080/10872981.2026.2756806)
Supplement: Supplementary file 1 [file ZMEO_A_2756806_SM6728.pdf]

Supplementary Information

Artificial Intelligence Hallucinations in Large Language Models for Medical Education and Healthcare: A Scoping Review of Detection, Mitigation, and Governance

Supplementary Table S1. PRISMA-ScR Checklist

Section	Item	PRISMA-ScR reporting item	Location in manuscript
Title	1	Identify the report as a scoping review.	Title
Abstract	2	Provide a structured summary including background, objectives, eligibility criteria, sources of evidence, charting methods, results, and conclusions.	Abstract
Introduction	3	Describe the rationale for the review in relation to existing knowledge.	Introduction
Introduction	4	State the review questions and objectives with reference to the Population–Concept–Context framework.	Introduction; Methods, Stage 1
Methods	5*	Indicate whether a protocol exists and provide registration details, if applicable.	it was not registered.
Methods	6	Specify eligibility criteria for sources of evidence, including years, language, context, and source type.	Methods, Stage 3
Methods	7	Describe all information sources, including databases, reference-list screening, and citation tracking.	Methods, Stage 2
Methods	8	Present the complete electronic search strategy for at least one database and provide full strategies in supplementary material.	Methods, Stage 2; Supplementary File 1
Methods	9	Describe the process used for screening and selecting sources of evidence.	Methods, Stage 3
Methods	10	Describe the data-charting process and whether charting was conducted independently by more than one reviewer.	Methods, Stage 4
Methods	11	List and define all data items extracted from included sources.	Methods, Stage 4
Methods	12*	If conducted, describe the rationale and methods for critical appraisal of included sources.	Methods, Stage 5
Methods	13	Describe methods used to handle and synthesize extracted data.	Methods, Stage 6

Results	14	Report the number of sources screened, assessed for eligibility, excluded, and included, ideally using a flow diagram.	Results; Figure 1
Results	15	Present characteristics of all included sources.	Tables 2 and 3
Results	16	If conducted, report results of critical appraisal.	Tables 2 and 3
Results	17	Present relevant findings from individual sources in relation to the review questions.	Tables 2 and 3
Results	18	Summarize and synthesize the results in relation to the review questions and objectives.	Results
Discussion	19	Summarize the main findings, themes, implications, and relevance to key groups.	Discussion
Discussion	20	Discuss limitations of the scoping-review process.	Discussion: Limitations and directions for future research
Conclusion	21	Provide an overall interpretation of the findings and implications.	Conclusion
Funding	22	Describe funding sources for the review and funding sources of included evidence, if reported.	Funding statement

* Optional PRISMA-ScR item.
