# Supplementary material for: Artificial intelligence hallucinations in large language models for medical education and healthcare: a scoping review of detection, mitigation, and governance
Source: Med Educ Online. 2026 Nov 6;31(1):2756806. doi: 10.1080/10872981.2026.2756806 (PMC13757853; doi:10.1080/10872981.2026.2756806)
Supplement: Supplementary file 2 [file ZMEO_A_2756806_SM6741.pdf]

Supplementary File 1. Full Search Strategies

Search date: July 31, 2026

Language restriction: English

Publication period: January 1, 2018, to July 31, 2026

PubMed/MEDLINE

Platform: PubMed

Records retrieved: n = 556

```
text
(("artificial intelligence hallucination*" OR "AI hallucination*" OR
"LLM hallucination*" OR "large language model hallucination*" OR
"generative AI hallucination*" OR "ChatGPT hallucination*" OR
"hallucination in large language models" OR confabulation OR
"fabricated information" OR "false citation*" OR "hallucinated citation*" OR
"fabricated citation*" OR "spurious citation*" OR "made-up citation*" OR
"invented fact*" OR "misinformation in AI" OR "factuality" OR "faithfulness")
AND
("artificial intelligence" OR AI OR "large language model*" OR LLM OR
"generative AI" OR ChatGPT OR GPT* OR "foundation model*"))
AND
(medicine OR medical OR "medical education" OR healthcare OR "health care" OR
clinical OR biomedical OR "health professions education" OR "medical
training")
AND
("2018/01/01"[Date - Publication] : "2026/07/31"[Date - Publication])
AND English[Language]
```

Scopus

Platform: Scopus

Records retrieved: n = 542

```
text
TITLE-ABS-KEY
(
  (
    "artificial intelligence hallucination*" OR "AI hallucination*" OR
    "LLM hallucination*" OR "large language model hallucination*" OR
    "generative AI hallucination*" OR "ChatGPT hallucination*" OR
    "hallucination in large language models" OR confabulation OR
    "fabricated information" OR "false citation*" OR "hallucinated citation*"
OR
```

```
    "fabricated citation*" OR "spurious citation*" OR "made-up citation*" OR
    "invented fact*" OR "misinformation in AI" OR factuality OR faithfulness
  )
  AND
  (
    "artificial intelligence" OR AI OR "large language model*" OR LLM OR
    "generative AI" OR ChatGPT OR GPT* OR "foundation model*"
  )
  AND
  (
    medicine OR medical OR "medical education" OR healthcare OR "health care"
OR
    clinical OR biomedical OR "health professions education" OR "medical
training"
  )
)
AND PUBYEAR > 2017
AND PUBYEAR < 2027
AND LIMIT-TO (LANGUAGE, "English")
```

IEEE Xplore

Platform: IEEE Xplore

Records retrieved: n = 525

Filters applied: Publication years 2018–2026; English language

```
text
(
  (
    "artificial intelligence hallucination*" OR "AI hallucination*" OR
    "LLM hallucination*" OR "large language model hallucination*" OR
    "generative AI hallucination*" OR "ChatGPT hallucination*" OR
    "hallucination in large language models" OR confabulation OR
    "fabricated information" OR "false citation*" OR "hallucinated citation*"
OR
    "fabricated citation*" OR "spurious citation*" OR "made-up citation*" OR
    "invented fact*" OR "misinformation in AI" OR factuality OR faithfulness
  )
  AND
  (
    "artificial intelligence" OR AI OR "large language model*" OR LLM OR
    "generative AI" OR ChatGPT OR GPT* OR "foundation model*"
  )
  AND
  (
    medicine OR medical OR "medical education" OR healthcare OR "health care"
OR
    clinical OR biomedical OR "health professions education" OR "medical
training"
  )
)
```

Web of Science Core Collection

Platform: Web of Science Core Collection

Records retrieved: n = 428

Timespan: January 1, 2018, to July 31, 2026

Language: English

```
text
TS=
(
  (
    "artificial intelligence hallucination*" OR "AI hallucination*" OR
    "LLM hallucination*" OR "large language model hallucination*" OR
    "generative AI hallucination*" OR "ChatGPT hallucination*" OR
    "hallucination in large language models" OR confabulation OR
    "fabricated information" OR "false citation*" OR "hallucinated citation*"
OR
    "fabricated citation*" OR "spurious citation*" OR "made-up citation*" OR
    "invented fact*" OR "misinformation in AI" OR factuality OR faithfulness
  )
  AND
  (
    "artificial intelligence" OR AI OR "large language model*" OR LLM OR
    "generative AI" OR ChatGPT OR GPT* OR "foundation model*"
  )
  AND
  (
    medicine OR medical OR "medical education" OR healthcare OR "health care"
OR
    clinical OR biomedical OR "health professions education" OR "medical
training"
  )
)
AND PY=(2018-2026)
AND LA=(English)
```

Manual Searching and Citation Tracking

The reference lists of all included sources were manually screened. Citation tracking of key included articles was also undertaken to identify potentially relevant sources. No additional sources identified through manual searching or citation tracking were included beyond those identified through database searching.

Supplementary Table S2. Number of Records Identified

Database	Records retrieved

PubMed/MEDLINE	556
Scopus	542
IEEE Xplore	525
Web of Science	428
**Total**	**2,051**
